# Management of Surgical Complications in Pediatric Kidney Transplantation

**DOI:** 10.3390/jcm15020779

**Published:** 2026-01-18

**Authors:** Maria P. Corzo, Sara K. Rasmussen, Jaimie D. Nathan

**Affiliations:** 1 Department of Abdominal Transplant and Hepatopancreatobiliary Surgery, Nationwide Children’s Hospital, 700 Children’s Drive, Columbus, OH 43205, USA; maria.corzo@nationwidechildrens.org (M.P.C.); sara.rasmussen@nationwidechildrens.org (S.K.R.); 2 Department of Surgery, The Ohio State University College of Medicine, 1645 Neil Ave., Columbus, OH 43210, USA; 3 Department of Pediatric Surgery, Nationwide Children’s Hospital, 700 Children’s Drive, Columbus, OH 43205, USA

**Keywords:** end stage renal disease, kidney transplant postoperative complications, vascular complications, urologic complications, graft nephrectomy

## Abstract

**Introduction:** Graft and patient survival after kidney transplantation in children has increased in the past decade; however, post-transplant surgical complications occur in up to 15.4% of recipients and pose a significant threat to graft survival. Due to anatomic discrepancies in children, kidney transplantation in this population is nuanced and requires meticulous planning. This narrative review summarizes the most common postoperative surgical complications following kidney transplantation in children. **Methods:** PubMed and Google Scholar were queried for full-text articles that reported pediatric kidney transplantation surgical complications and their management following kidney transplantation. **Results:** Vascular complications can occur in approximately 1.3–13.8% of cases and are the leading cause of graft nephrectomy, with arterial stenosis and venous thrombosis being the most common. Urologic complications occur in 1.3–30% of patients and are more frequent in children due to pre-existing genitourinary abnormalities prior to transplantation. Vesicoureteral reflux is the most common urologic complication. **Discussion:** Surgical complications following kidney transplantation in children continue to significantly affect graft viability. Ultimately, meticulous surgical techniques and close postoperative surveillance are critical to mitigating the risk of allograft nephrectomy. Prospective studies focused on best surgical practice, techniques, prevention, and postoperative care in pediatric kidney transplant recipients are needed.

## 1. Introduction

In pediatric patients, end stage renal disease (ESRD) has a profound negative impact on adequate growth, puberty, nutrition, and quality of life, and also impacts appropriate physical and social development [1,2,3]. Pre-emptive transplantation is currently the treatment of choice, since dialysis does not mitigate the full extent of morbidities associated with renal failure in children [4,5,6,7]. Furthermore, dialysis has been associated with greater mortality, decreased quality of life, decreased kidney transplant success, and greater cost in comparison to kidney transplantation [5,6,7].

Pediatric kidney transplantation has steadily increased from <2% of total kidney transplants in the United States in 2022 [8] to 2.75% in 2025 [8,9]. Data from the Organ Procurement and Transplantation Network (OPTN) reports a total of 625,413 kidney transplants in the United States since 1988 [9]. More specifically, 27,983 (4.5%) were pediatric kidney transplants, of which 16,763 were performed in the 11–17-year age group and 118 in the <1 year age group.

Over the last decade, graft survival has increased [10], and current pediatric kidney transplant outcomes are similar to adult outcomes [4,8,11]. Data from the 2023 OPTN reports an overall 5-year patient survival rate of 97.3% for pediatric deceased donor kidney transplant (DDKT) recipients and 98.6% for pediatric living donor kidney transplant (LDKT) recipients, respectively [12]. One-year, three-year, and five-year graft survival rates for pediatric DDKT recipients were 97.6%, 93.6%, and 86.9%, respectively, while pediatric LDKT recipients had one-year, three-year, and five-year graft survival rates of 99.1%, 96.4%, and 93.1%, respectively.

Despite the overall improvement in patient and graft survival rates, surgical complications in this specific population have been reported to occur in 4–15.4% of cases [13,14]. Furthermore, children <15 kg that undergo kidney transplantation have been noted to have a greater risk for surgical complications of up to 35%, of which 26% have required reoperation [15]. Given that one of the primary etiologies of ESRD in children is congenital anomalies of the kidney and urinary tract (CAKUT), they more frequently require urologic procedures prior to transplantation. In this context, allografts are more susceptible to post-transplant urologic and vascular complications, such as vesicoureteral reflux (VUR), ureteral stricture, and vascular thrombosis, requiring reoperation [8,14,16,17]. The most common postrenal transplant complications are of vascular and urologic origin [15], and in over 50% of those who undergo graft nephrectomy, the cause is related to graft-threatening complications [18]. Moreover, graft nephrectomy poses significant technical challenges and is associated with increased peri- and postoperative morbidity and mortality ranging from 1 to 39% [18]. Patients who have undergone graft nephrectomy followed by re-transplantation have worse overall outcomes in comparison to recipients who do not undergo re-transplantation [11,18].

Considering this, kidney transplantation in children requires meticulous surgical planning and execution that takes into consideration the anatomic discrepancies that are nuanced to this specific population. Moreover, adopting standardized perioperative and postoperative practices facilitates early recognition and more efficient management of post-transplant complications.

Recognizing the critical threat that post-kidney transplant complications pose on allograft viability, the aim of this narrative review is to summarize the management of the most common surgical complications following kidney transplantation in children. We have focused on underscoring the critical roles of surgical expertise, perioperative vigilance, and the role of timely recognition of warning signs to optimize patient and graft outcomes. We describe patient-specific risk factors and clinical presentations, and we evaluate management strategies and approaches for prevention of these complications.

## 2. Methods

This narrative review was performed following the SANRA guidelines [19]. Literature was searched using PubMed and Google electronic databases. The references of selected manuscripts were reviewed and included if they met inclusion criteria. Inclusion criteria were full-text articles published in English on patients ≤18 years old who underwent living donor or deceased donor kidney transplantation and reported on surgical complications post-transplantation. No limitations were imposed on the type of studies included or year of publication. The primary sources of data were pediatric studies; in instances where data was insufficient in children, additional data from the adult population was used to complement. Epidemiology, risk factors, and outcomes are primarily based on pediatric data, unless specified. Search terms include a combination of keywords and Boolean operations, such as (“surgical complications” AND “kidney transplant”) OR (“renal transplant” AND “children” OR “pediatric populations”), (“surgical complication after renal transplantation”) AND (“pediatric populations”), (“vascular complications”) AND (“kidney transplant”) AND (“children” OR “pediatric population”), and (“urologic complications”) AND (“kidney transplant”) AND (“children” OR “pediatric population”). Data were synthesized to summarize epidemiology, clinical manifestations, diagnosis, and management of the most common surgical complications following pediatric kidney transplantation.

## 3. Vascular Complications

Vascular complications after kidney transplantation are the most common surgical complications in children, with a reported risk ranging from 1.3 to 13.8% [2,8,10,11,14,20]. These complications include arterial stenosis, venous thrombosis, arterial thrombosis, and arterial anastomotic aneurysm, of which arterial stenosis and venous thrombosis are the most common.

### 3.1. Vascular Stenosis

#### Arterial Stenosis

Arterial stenosis has been reported in up to 1–35% of adult kidney transplants [21,22]; however, data in children is scarce with a reported incidence of 5–9% [14,23]. Kinking or trauma to the artery during graft procurement or implantation, vascular rejection, suturing technique, external compression (related to perinephric collections), atherosclerosis, or cytomegalovirus (CMV) infection are potential causes of post-transplant arterial stenosis [18,21,23,24]. Stenosis becomes clinically significant when intraluminal diameter is narrowed by more than 50% [21]; however, there is no clear consensus regarding the degree of renal arterial narrowing that requires invasive treatment [22,23]. Manifestations are secondary to the progression of the stenotic area and can arise at any moment postoperatively, leading to hypertension, graft dysfunction, and even graft loss [21]. Patients may be asymptomatic or present with aggravated or refractory arterial hypertension and serum creatinine elevation in the absence of hydronephrosis, rejection, or infection [21,23,25].

Postoperative interval imaging with Doppler ultrasound is fundamental in identifying asymptomatic cases or when stenosis is initially suspected. CT or MR angiography are the preferred method of imaging; however, CT and MR without angiography can also confirm diagnosis and aid with surgical planning [22,23]. Stenosis most commonly occurs at the site of vascular anastomosis; however, it can also present pre-anastomotic and post-anastomotic, or with diffuse arterial stenosis [22].

**Figure 1:**
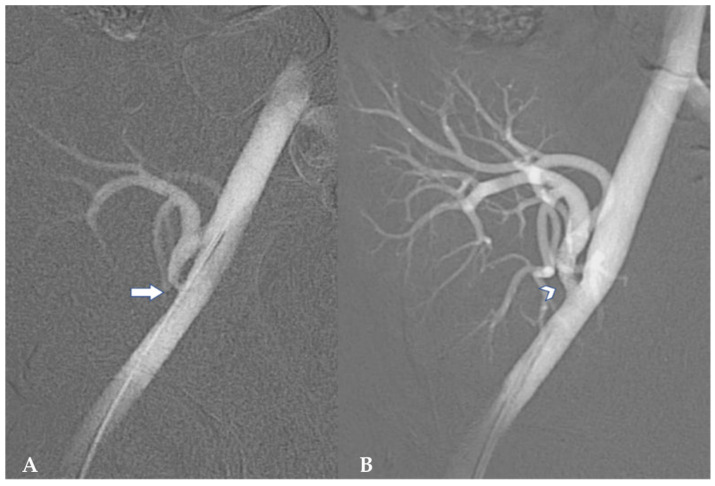
Percutaneous transluminal angiography for management of renal artery stenosis. (**A**) Renal artery stenosis at site of anastomosis (arrow). (**B**) Resolution of stenosis after endovascular stent deployment (arrowhead). Image reused from “Kidney transplant artery and vein stenting: 15-year follow-up”, by Peregrin JH et al., published by CVIR endovascular, accessed on 18 December 2025, licensed under CC BY 4.0 (http://creativecommons.org/licenses/by/4.0/) [27]. Minor modifications were made to the title and descriptive captions for panels (**A**,**B**). Image was cropped and pixels were modified.

Treatment ranges from medical management, interventional radiology intervention, or open surgical repair. Low-dose, short-acting angiotensin converting enzyme inhibitors are the first line of medical treatment for hypertension related to arterial stenosis [23]. However, in the presence of high blood pressure that does not respond to pharmacologic treatment or if kidney function deteriorates, further treatment is necessary [23]. Indications for revascularization of renal artery stenosis in renal transplant recipients are lacking; however, it is widely accepted that luminal narrowing of <50% is not considered hemodynamically relevant [21,23]. Percutaneous transluminal angiography (PTA) with balloon dilation, with or without endovascular stent deployment, is the first line of management [21,22,25] (Figure 1) and has a success rate of 70–90% in adult and pediatric patients [21]. Serum creatinine levels normalizes within 3–5 days, and hypertension has been reported to resolve in up to 63–83% of adult patients [21]. Single institution experience with 10 pediatric patients undergoing PTA for renal artery stenosis (9 of 10 with >85% luminal narrowing and 1 with double stenosis and 50–75% luminal narrowing) reported significant improvement in graft function, a 14% rise in creatinine clearance at 30–60 days, and a total success rate of 80% with no complications [23]. After PTA, 80% of patients had at least 50% luminal gain and no re-interventions were reported. At a median follow-up of 4.1 years, all patients had functioning grafts with no PTA-related complications. Regular Doppler US imaging (monthly for 6 months and then twice a year) is advocated for surveillance, as transplant renal artery stenosis can be clinically silent. Moreover, another retrospective study from 2025 reported a post-transplant renal artery stenosis rate of 7.8% in children [26]. Five of seven patients underwent balloon angioplasty alone, and the remaining underwent stent placement. The reported overall success rate was 83.3%, with an overall restenosis rate of 28.6%, and 40% in those who underwent balloon angioplasty alone. Graft function improved with a mean creatinine clearance rate increase of 34% over two years and a 10% reduction in median arterial pressure. In less than 4% of adults who undergo PTA, arterial dissection, rupture and even thrombosis have been reported [21]. If interventional radiology management fails, the stenosis is severe, or PTA is contraindicated, open surgical management may be required [14,21,23,25].

### 3.2. Vascular Thrombosis

Vascular thrombosis (VT) is a feared complication, as almost 100% of cases lead to graft loss, and is the main indication for graft nephrectomy during the early postoperative period [11]. A metanalysis from 2025 evaluating the benefits of thromboprophylaxis in pediatric kidney transplant recipients identified a pooled rate of graft loss secondary to graft thrombosis of 78% [28]. However, rates of allograft loss related to venous and arterial thrombosis have been reported to occur in up to 100% of cases [14]. The overall reported incidence of vascular thrombosis after kidney transplantation in children is between 2 and 10% [6]. Recipient age < 2 years, donor age < 6 years, cold ischemic time > 24 h, prior peritoneal dialysis, hypoperfusion, delayed graft function, multiple donor vessels, and known prothrombotic abnormalities have been identified as risk factors [3,20]. High index of suspicion for VT is warranted in patients with oliguria/anuria and hemodynamic changes in the setting of a previously functioning graft [20].

Post-transplant vascular thrombosis has been associated with technical complications, such as torsion, kinking, and compression, or endothelial damage secondary to excessive traction and manipulation [20]. Furthermore, inherent vessel caliber discrepancies between kidneys obtained from very young or adult donors and a pediatric recipient can further increase the risk of vascular thrombosis [7]. Intraoperatively, evaluating the best choice for implanting the mismatched donor vessels to larger vessels, like the aorta and inferior vena cava, can mitigate this risk [29]. Specific attention to the renal artery and vein during dissection, handling, positioning, and anastomosis is vital. More specifically, caution is warranted when evaluating the relation of vessel length, anatomic positioning of the renal artery and vein, and the arterial and venous anastomoses, so as to avoid any potential risk for obstruction secondary to surgical technique [17]. Prior to closure, confirming adequate graft and vessel position aids in addressing any potential causes of external obstruction that can predispose patients to venous hypertension and renal vein thrombosis [17].

A retrospective study from Spain assessed the impact of introducing a standardized protocol for the prevention, early detection, and treatment of vascular complications [20]. Following the implementation of this protocol, 100% of patients who underwent immediate surgical intervention for vascular complications successfully recovered graft function, compared to a 75% recovery rate before the protocol was established. This protocol involved postoperative thromboprophylaxis with aspirin (ASA) and heparin, maintaining a central venous pressure (CVP) > 10 mmHg intraoperatively and, daily Doppler ultrasound for the first 4 days post-transplantation. Special intraoperative attention to avoid excessive vessel handling and optimization of renal vein length and anatomic location were also part of the protocol.

#### 3.2.1. Venous Thrombosis

Renal vein thrombosis in children has a risk of 0.5–4%, and patients < 4 years old with lower body weight carry the greatest risk [8,18]. This complication has an early postoperative onset and is among the most common etiologies of acute graft loss after kidney transplantation [24,30]. A retrospective multicenter French study evaluating the management of renal vein thrombosis in adults reported that three of five cases that presented with venous thrombosis required graft nephrectomy [30]. Etiology ranges from prolonged ischemia, hypovolemia, endothelial damage, surgical difficulty, venous compression, and underlying hypercoagulable states [24]. However, if cases are identified intraoperatively, the most common findings are a twisted renal vein, iliac vein strangulation, long vein length, or caliber disparity between donor and recipient vessels [30]. Intraoperative findings include a mottled or necrotic allograft. Early phases of acute venous thrombosis lead to renal allograft enlargement but as thrombosis duration increases, the kidney shrinks [24]. Acute onset of pain, oligoanuria, kidney dysfunction, ipsilateral lower extremity swelling, and low-grade fever are commonly presenting symptoms [21,30]. Furthermore, embolic and hemorrhagic complications can ensue secondary to thrombosis, increasing patient morbidity and mortality [30].

**Figure 2:**
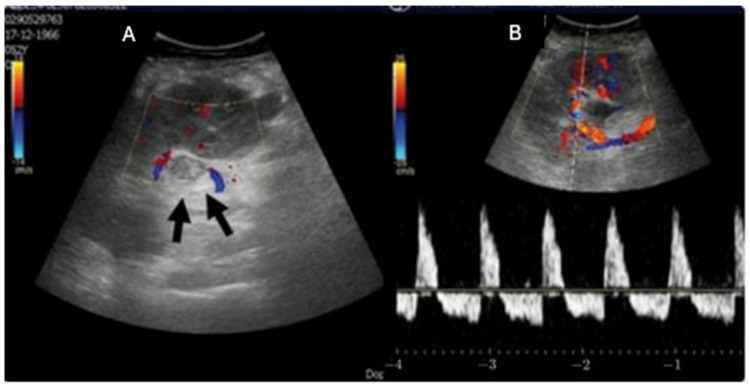
Thrombosis of renal vein demonstrated by color Doppler ultrasound. (**A**) Enlarged left renal vein with hypoechogenic content, without Doppler signal (black arrow). (**B**) Arterial reversal of diastolic flow. Image reused from “Unexpected success in early post-transplantation renal vein thrombosis: A case report and literature review”, by Santos JE et al., published by Clinical Nephrology Case Studies, accessed on 18 December 2025, under Public Domain (PDM 1.0) (https://creativecommons.org/publicdomain/mark/1.0/) [31]. Minor modifications were made to the title and descriptive captions. Labels (**A**,**B**) are new to this figure. Numbers on the scale were modified, but interpretation is unchanged.

**Figure 3:**
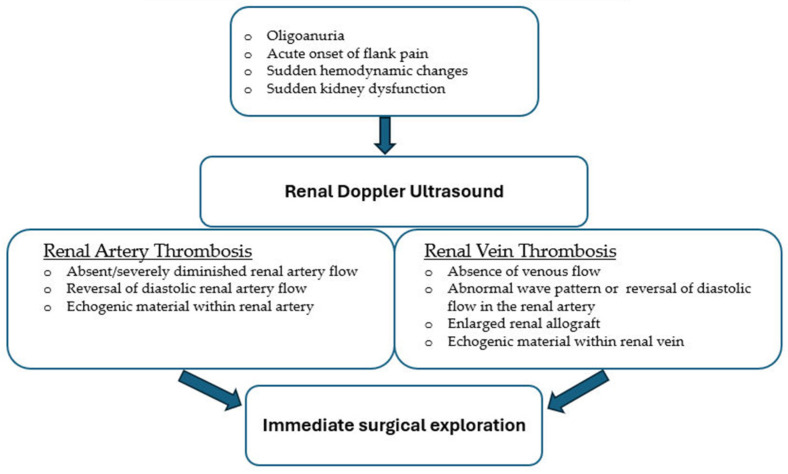
Management of Post-Transplant Vascular Thrombosis.

A high index of suspicion is critical for timely detection. Doppler ultrasound, CT or MR angiography provide confirmation when complete absence of venous flow or renal graft enlargement are present, amongst other findings (Figure 2) [21]. Notably, significantly concerning features on Doppler US, described above and shown in the management algorithm in Figure 3, should be an indication to proceed with immediate re-exploration, as time is crucial in attempts to salvage the graft. Standard use of intraoperative ultrasound in certain institutions accounts for early identification of venous thrombosis and has resulted in greater rates of graft salvage [30]. Immediate surgical venous anastomotic repair can mitigate substantial graft damage and lead to more favorable outcomes [30]. Figure 3 summarizes diagnostic and therapeutic pathways for the management of venous thombosis.

Emergent surgical revision entails thrombectomy via venotomy or thrombectomy with anastomotic revision. Alternatively, the graft may be removed, flushed with preservation solution, and reimplanted if the kidney is salvageable [30]. A necrotic graft is indication for graft nephrectomy [30]. Caution is warranted, as long-term graft outcomes in the setting of acute venous thrombosis that was treated and resulted in a viable graft, can be challenging. The extent of microvascular, tubular and cortical necrosis, and ischemia–reperfusion damage make it difficult to know if there will be complete graft recovery or if progression to graft dysfunction will ensue [30]. Ultimately, preventative intraoperative tactics and high index of suspicion are the cornerstones to salvaging grafts, as venous thrombosis commonly leads to graft nephrectomy [21].

#### 3.2.2. Arterial Thrombosis

Although documented as rare, with a reported incidence ranging from 0.5 to 12% in the overall population [21] and 2% in children [14], this complication requires timely diagnosis to avoid graft loss during the early post-transplant period [21]. Increased risk has been associated with the presence of multiple renal arteries, kinking, hypotension, hypercoagulability-predisposing diseases (e.g., nephrotic syndrome and systemic lupus erythematous), and technical difficulties during operation [21,24,25]. Acute decrease in urine output and onset of renal dysfunction are the most common clinical manifestations [21].

Color Doppler ultrasound depicts arterial thrombosis and establishes absent flow into both the main and intrarenal arterial branches [21,25]. Although catheter-based angiography and MR angiography are considered as the gold standard in diagnostic confirmation [21], concerning features on Doppler US are criteria enough for immediate surgical exploration (Figure 3). Immediate surgical exploration and revascularization via thrombectomy, with anastomotic revision as needed, are indicated, but most cases result in graft nephrectomy [20,21,25]. The current role of interventional radiology for arterial thrombosis is not clear, as catheter-directed thrombolytics are not recommended during the first 10–14 days post-transplantation due to the risk of major postoperative bleeding [21]. Figure 3 summarizes diagnostic and therapeutic pathways for the management of arterial thrombosis.

Wound closure requires special consideration, as thrombosis causes edema and increased tension, which can lead to further complications, such as abdominal and renal allograft compartment syndrome. Gondry et al. endorse the use of non-absorbable mesh to aid in minimizing wound tension. However, progressive wound approximation with definitive closure by day 3–4 after adequate graft function is confirmed has also been reported [20].

#### 3.2.3. Thromboprophylaxis

The role of thromboprophylaxis in the setting of pediatric kidney transplantation is unclear, and its use and potential risk have not been fully elucidated. A 2025 systematic review evaluating the benefits of prophylactic anticoagulation in pediatric kidney transplant recipients, with a total of 25 observational studies, reported that 64% received thromboprophylaxis regardless of thrombotic risk, and 36% received thromboprophylaxis only if deemed high risk [28]. Thromboprophylaxis was associated with a lower risk of graft thrombosis (4% vs. 12%). Furthermore, the use of thromboprophylaxis was not associated with a significantly higher risk of bleeding resulting in surgical re-exploration or graft loss, and no fatal bleeding events occurred in this group. Notably, thromboprophylaxis regimens for the 1659 patients varied substantially. In 38.2% of patients, heparin-based thromboprophylaxis was used, while 12.2% received aspirin, 0.6% received dipyridamole, and 49% received a combination of agents. These regimens were initiated intraoperatively, or within the first week postoperatively, and were continued for up to a 1 year after kidney transplantation. Although this systematic review provides reassuring data for the use of thromboprophylaxis in pediatric kidney transplant recipients, heterogeneity among distinct study outcomes, the use of multiple or distinct types of anticoagulants, and discrepancies regarding initiation, duration, and dosage limit the generalizability of the study. As such, the appropriate timing, preferred antithrombotic medication, and duration remain inconclusive.

### 3.3. Arterial Aneurysm

**Figure 4:**
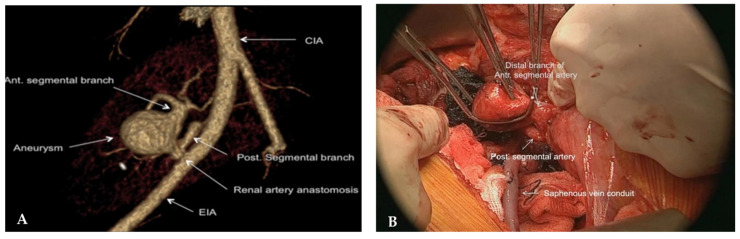
CT angiogram 3D reconstruction (**A**) and intraoperative image (**B**) demonstrating renal artery aneurysm. (**A**) Arterial aneurysm of the anterior segmental branch seen on 3D CT angiogram. (**B**) Intraoperative image of renal artery aneurysm involving the anterior segmental branch. CIA: common iliac artery, EIA: external iliac artery. Image reused from “Successful repair of transplant renal artery aneurysm (TRAA)”, by Ho CERH et al., published by BMC Urology, accessed on 18 December 2025, licensed under CC BY 4.0 and CC0 1.0 Universal (http://creativecommons.org/licenses/by/4.0/ and https://creativecommons.org/publicdomain/zero/1.0/) [32]. These figures were cropped and merged from the original article to create this new single figure. Labels (**A**,**B**) were not in the original figure. Minor modifications were made to the title and descriptive captions.

Arterial aneurysm after kidney transplantation in children and adults is very rare (≤1%) and can manifest as an early or late postoperative complication [14,21,32]. Technical difficulties during operation, infections, and immunological conditions can lead to aneurysm formation [21]. True aneurysms can be asymptomatic, but patients can experience abdominal pain with or without hypertension or hemorrhage [21,32]. Complications can be catastrophic, including aneurysm rupture, renal artery thrombosis, and arterial dissection, leading to renal infarction [32]. Color Doppler ultrasound, CT angiography, or MR angiography can be used to confirm the diagnosis (Figure 4) [33]. Notably, workup is required to evaluate for an infectious etiology, which must be treated accordingly [33].

Small or asymptomatic pseudoaneurysms may not need surgical intervention and can be closely followed. However, if greater than 2.5 cm, if there is rapid growth, or if a mycotic aneurysm is identified, surgical or interventional radiology management is indicated [33]. Management options include open surgical repair, percutaneous thrombin injection under US guidance, and endovascular treatment with stenting or embolization [33]. Surgical intervention is often preferred and entails aneurysm resection with or without arterial reconstruction using autologous or synthetic grafts, while graft nephrectomy may be required for management of a mycotic aneurysm [14,21,32]. Endovascular treatment with coil embolization with or without stenting has been successful in treating adults with renal artery pseudoaneurysms [33]; however, its implementation in the pediatric population remains to be defined.

## 4. Urologic Complications

Post-transplant urologic complications in children have a combined incidence ranging from 3.1 to 30% [14,34,35]. Children more frequently develop these complications due to the presence of pre-existing genitourinary abnormalities and the technical challenges associated with these [35]. Pre-existing urologic malformations have been associated with greater risk for urological complications [14,36]. Urinary leak and early ureteral stenosis typically present during the first month after transplantation. Late complications include urinary tract infections and late ureteral obstruction related to fibrosis, infection, and rejection [18].

The use of temporary ureteral stents in kidney transplantation remains controversial in both adult and pediatric populations. In adults, stents have been reported to be protective against stenosis or leakage and have also been associated with a 1.5–7% decrease in the risk of urologic complications [18]. An electronic survey sent out to pediatric transplant centers worldwide by the European Society for Paediatric Nephrology (ESPN) in 2022 evaluating the diagnostic and therapeutic management of vesicoureteral reflux in pediatric kidney transplantation identified that 90% of centers placed ureteral stents at time of transplantation [37]. However, studies have reported ureteral stents to be associated with an increased risk of urinary tract infections [38] and BK viremia and viruria [38,39]. Furthermore, several retrospective studies in children reported no significant difference on the rates of ureteral stenosis or leakage between patients that had ureteral stent placement during kidney transplantation versus those that did not [35,38,40]. Currently, the use of stents differs among institutions; however, when being used, the recommendation is for early postoperative removal to prevent infectious complications [16].

### 4.1. Vesicoureteral Reflux

Vesicoureteral reflux (VUR) is often noted as one of the most common urologic complications after kidney transplantation, and although its true rate post-transplant is difficult to estimate, it has been reported to occur in 1.7–60% of cases in children [10,16,36,41]. It is usually asymptomatic, and its true incidence in children is challenging to determine, partly due to that fact that a portion of this population is not continent, making it difficult to evaluate and compare to adult data [18]. Furthermore, routine voiding cystourethrogram (VCUG) prior to, or after transplantation, is not standard practice in most centers [18], and true rates of VUR may be underreported. The previously mentioned electronic survey sent by the EPSN in 2022 identified that VUR screening post-transplantation was only performed in 7% of transplant centers [37]. Additionally, diverse surgical techniques while performing ureteroneocystostomy during transplantation [18] can also introduce heterogeneity in reported outcomes.

Urinary tract infections (UTIs) can be the presenting manifestation; therefore, initial treatment includes addressing the acute infection with antibiotics [36]. UTIs are associated with graft scarring, acute allograft dysfunction, increased risk for acute rejection, and a greater risk for long-term graft functional decline [16]. VUR can be suspected in post-transplant kidney recipients who develop febrile urinary tract infections, have persistent hydronephrosis or ureteral dilation of the transplant kidney, especially in patients with previous bladder dysfunction or bladder surgery [37,42]. VCUG is most commonly used for diagnosis, however voiding urosonography may be helpful [37].

Surgical treatment is not always indicated to manage VUR. However, a setting in which VUR may need to be addressed surgically is in the presence of recurrent graft pyelonephritis that leads to subsequent graft damage [16]. Generally, when VUR is symptomatic or in the presence of UTI recurrence, initial nonsurgical management may include endoscopic polymer injections into the opening of the ureter in the bladder, which acts as a valve and aids in reducing VUR [10,14]. Although less invasive than surgical revision of the ureteroneocystostomy, data regarding recurrence risk and secondary ureteral obstruction, have limited its use [16]. When infections and pyelonephritis are recurrent, or ureteral obstruction is evident, surgical repair with ureteral reimplantation may be necessary [36]. Given the proximity of the ureters to the iliac vessels, the characteristics of the primary ureteral anastomosis, the ureteral vasculature, and the presence of fibrosis, repair can be challenging and can result in morbidity [16,41]. Multiple studies have reported a significant reduction in the total number of UTIs and recurrent pyelonephritis episodes after ureteroneocystostomy revision, which not only positively impacts the graft, but patient quality of life and medical costs [16]. Furthermore, Campbell et al. reported a durable decrease and resolution of VUR symptoms in children who had ureteroneocystostomy revision [16]. Additionally, a systematic review evaluating the success rates of revision ureteroneocystostomies after pediatric kidney transplantation reported a success rate of 91% (range: 40–100%) and a lower complication rate than endoscopic management [41].

There are numerous ureteroneocystostomy techniques during initial transplantation that can ultimately influence urologic complication rates. However, in the pediatric population, it is unclear whether any technique is favored [18]. Some surgical techniques include intravesical or extravesical ureteral implantation and refluxing or anti-refluxing techniques. Currently, anti-reflux techniques for ureteral implantation are the gold standard in adult kidney transplantation [43]. Post-kidney transplantation VUR in pediatric patients who undergo reimplantation using anti-reflux techniques has a reported occurrence of up to 60%, whereas in the absence of anti-reflux techniques, VUR has been reported in up to 79% of patients [41]. Importantly, anti-reflux techniques can increase operative time and the risk for ureteral obstruction [16,18].

### 4.2. Ureteral Obstruction

Ureteral obstruction occurs in up to 8% of pediatric kidney transplants [18,36]. Ureteral stenosis, ureterovesical junction obstruction, and external ureteral compression (perinephric collection, adhesions) are amongst the primary etiologies [36].

#### 4.2.1. Ureteral Stenosis

Ureteral stenosis has been reported in up to 5% of pediatric kidney transplants and can have distinct etiologies based on its timing [14]. Early stenosis, defined as within 3 months, is most likely associated with surgical technique or ureteral blood supply compromise during operation [25]. Stenosis at 6 months or later postoperatively is more likely secondary to infection, fibrosis, progressive vascular disease, or rejection [25]. Persistent hydronephrosis, renal dysfunction, frequent UTI or local pain and distention can be indicative of ureteral stenosis that requires treatment [25].

**Figure 5:**
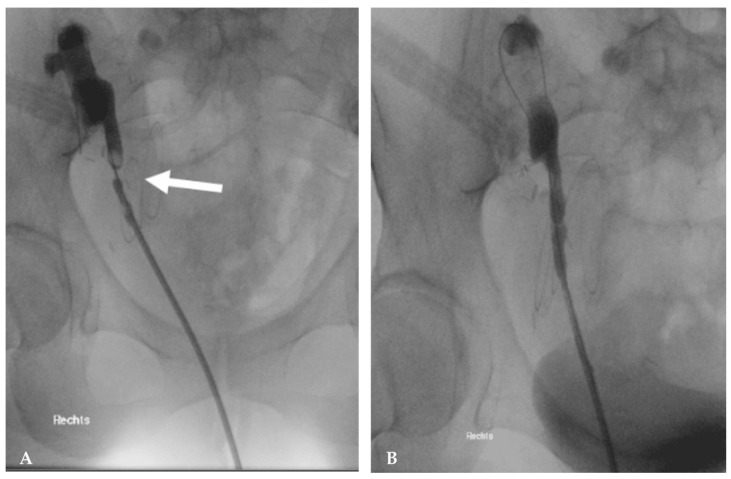
Interventional balloon dilation of proximal ureteral stricture. (**A**) Ureteral stricture (arrow). (**B**) Regular contrast passage after dilation. Image reused from “Minimal-invasive management of urological complications after kidney transplantation”, by Deininger S et al., published by International Urology and Nephrology, accessed on 18 December 2025, licensed under CC BY 4.0 (http://creativecommons.org/licenses/by/4.0/) [43]. Changes were made to only include (A) and (D) portions of the original figure, and (D) was relabeled to (B). Minor modifications were made to the title, image labels and descriptive captions. Image was cropped and pixels were modified.

**Figure 6:**
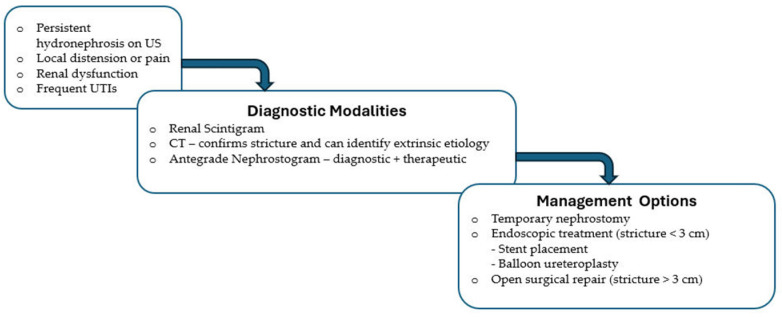
Management of Post-Transplant Ureteral Stenosis.

Initial evaluation consists of US that can demonstrate hydronephrosis, followed by renal scintigram, and antegrade nephrostogram to confirm obstruction and plan treatment options [35,44]. CT imaging further delineates the location of ureteral obstruction and identifies external causes, such as perinephric fluid collections. If the ureteral stent placed at transplant is obstructed, the stent can be replaced via cystoscopy to alleviate obstruction. Management options for strictures include percutaneous nephrostomy tube with antegrade pyelogram and stent placement [14,25], balloon ureteroplasty (Figure 5), holmium laser endoureterotomy, or open surgical repair [25]. Stricture length helps stratify whether endoscopic management (<3 cm) or surgical reconstruction (>3 cm) is indicated [25,43]. Ureterovesical reimplantation can be performed if previous anastomosis is faulty, or if ureteral necrosis is identified during surgical exploration [14]. In the setting of ureterovesical junction obstruction, anastomotic revision with or without resection, intravesical reimplantation, or ureteroureterostomy into the native ureter may be performed [36]. Figure 6 summarizes the diagnostic and therapeutic pathways for the management of ureteral stenosis.

#### 4.2.2. Ureteral Stenosis Due to BK Infection

In the context of post-kidney transplant ureteral stenosis, it is equally important to take into consideration non-technical causes, such as BK virus infection. BK viruria in pediatric renal transplant patients has a median onset of 3 months post-transplant and has been reported in up to 63% of patients, while BK viremia has been reported in up to 21% [31]. Ureteral stenosis secondary to BK virus infection usually presents late postoperatively and has a prevalence of 2–6% in adult kidney transplant recipients [45]. In children, a single institution reported two cases (1%) of ureteral stenosis secondary to BK infection or reactivation [46]. BK virus remains latent and asymptomatic in the uroepithelial cells of immunocompetent patients; however, in the setting of immunosuppression, BK virus can reactivate, and infection can quickly disseminate [39]. The use of ureteral stents during kidney transplantation, aggressive immunosuppression, pediatric age, and a high degree of human leukocyte antigen mismatch have been identified as risk factors [39]. Studies have reported a 4-fold increased risk of BK virus in both adult and pediatric kidney transplant recipients that have ureteral stents [13,39]. While stent use has been reported to reduce the risk of stenosis, obstruction, and urinary leaks after kidney transplantation, it is thought that the mechanical trauma induced by ureteral stents can lead to erosions, ulcerations, and inflammatory reactive changes that can also favor vesicoureteral reflux and promote BK virus pathogenesis [39].

BK nephropathy manifests as hemorrhagic cystitis, ureteral stenosis, and interstitial nephritis, and can mimic symptoms of acute rejection [47,48]. Ureteral stenosis secondary to BK infection can lead to segmental ischemia and fibrosis of the epithelial lining of the ureter, causing irreversible ureteral stenosis, which has only been reported in the setting of kidney transplantation [45,48].

Initial treatment entails achieving control of BK infection by decreasing immunosuppression, and if necessary, percutaneous nephrostomy can be performed while infection and inflammation clear [31,45]. As BK infection subsides, workup to assess ureteral stenosis with renal scintigraphy and antegrade nephrostogram, confirm site of stenosis and help define further management. Late developing ureteral obstruction, often related to fibrosis and rejection, are more often located proximally and have a worse response rate to percutaneous therapy (response rate of 16–33%) [44]. Treatment options to address ureteral stenosis include antegrade stent placement and open surgical ureteral repair, as ischemia and irreversible ureteral fibrosis require definitive treatment.

### 4.3. Urinary Leak

Urinary leaks have been reported in 2–3% of kidney transplants in pediatric recipients [10,14,36] and can occur secondary to anastomotic dehiscence, ureteral necrosis, or mechanical injury to the ureteral wall [36]. Clinical presentation includes localized pain and swelling, associated with increased serum creatinine, oliguria, or signs of infection. Notably, leaks can also manifest through abdominal drains or at the incision and can predispose patients to delayed wound healing and perinephric infections [49].

Ultrasound is the initial method used for diagnosis; however, CT scan or cystogram, identifies the location of the leak [49]. The most common site of leak is at the distal portion of the ureter or at the site of the ureteroneocystostomy [38]. Antegrade pyelogram during nephrostomy tube placement remains the gold standard for diagnosis, although needle aspiration of fluid collection for biochemical analysis is often the first step in evaluation [49]. After nephrostomy tube placement, interval imaging with US can be used to evaluate for resolution or progression that warrants surgical repair [14,25]. Conservative leak management entails percutaneous nephrostomy followed by antegrade stenting, and placement or exchange of Foley catheter [49]. Surgical exploration is indicated if a leak persists, infection control is warranted, or if necrosis of the ureter is suspected [49]. Surgical management includes the use of double-J stenting, ureteroureterostomy into the native ureter, and ureteral reimplantation [14,25,49]. When a urinary fistula develops as a manifestation of a leak, ureteral reimplantation or ureteroureteral anastomosis with the native ureter are definitive surgical options [10].

## 5. Surgical Site Infections

Solid organ transplant recipients carry a greater risk for surgical site infections (SSIs), which represent a threat to graft survival and an important cause of increased morbidity and mortality [50,51,52]. Rates of surgical site infections differ according to the type of operation, but in solid organ transplantation, SSIs have a reported incidence that ranges between 3 and 53% [52]. In pediatric kidney transplantation, wound infections have been reported in up to 3.2% of cases [13], and in adults, the incidence ranges between 3 and 11% [52]. Although renal transplant recipients carry the lowest SSI risk among solid organ transplant recipients, the incidence is not negligible [52]. Deep incisional and organ/space infections have been associated with increased readmissions and worse overall outcomes [51].

Technical complexity, prolonged operative duration, postoperative hematoma, urinary leak, and immunosuppression in pediatric organ transplant recipients predispose this population to a greater risk of surgical complications, including an increased risk of post-transplant infections [50]. Prevention of SSIs includes minimizing operative time, optimizing sterility and surgical technique, alongside maintaining intraoperative and postoperative normothermia, oxygenation, and glycemic control [52]. Antimicrobial prophylaxis with the narrowest spectrum agent, for the shortest duration that provides coverage for Gram-positive organisms, enterococcus species, Gram-negative uropathogens, and Candida, is indicated. In the setting of kidney transplantation, antibiotic prophylaxis with a first-generation cephalosporin for less than 24 h is recommended [50].

Localized pain, swelling, erythema, purulent drainage, and fever are the most common presenting symptoms. Superficial SSIs are initially managed with pressure irrigation or sharp excision to remove non-viable tissue and exudate, after adequate visualization of the incision confirms that no additional dehiscence, fistula, or drainage is concomitantly occurring [52]. Empiric antibiotic therapy should cover the most predominant microorganisms and should be tailored to clinical response of the patient and to Gram stain and wound culture results [52]. In the setting of deep incisional and/or organ/space SSIs, treatment is more aggressive and requires antibiotic administration, percutaneous or surgical drainage, surgical debridement, and source control [52].

## 6. Lymphocele

Lymphoceles are the most common postoperative lymphatic complication and are the most common fluid accumulation after kidney transplantation, with an incidence ranging from 0.5 to 22% in children [18,36,53]. They most often manifest within the first week after transplantation, typically surround the graft, and are secondary to transection and inadequate ligation of lymphatic channels during transplant operation [18,25,36,54]. Certain identified risk factors include older recipient age, high body mass index (BMI), number of transplant operations, diabetes, coagulation abnormalities, immunosuppressive medications, and acute rejection [18,25,54]. Deteriorating graft function can ensue, secondary to extrinsic compression of renal vessels, the ureter, or the bladder leading to bladder outlet obstruction. Presenting signs and symptoms include flank or abdominal pain, inguinal or lower extremity edema, and deep vein thrombosis [18,36].

Ultrasound and fluid aspiration analysis are the cornerstone to lymphocele diagnosis [54]. Although initial management is surveillance, as most are asymptomatic and spontaneously resolve, if large enough, complications can arise [14,18,52]. Large and symptomatic lymphoceles are treated with needle aspiration and percutaneous drainage. However, if this fails, surgical fenestration and peritoneal drainage is indicated [14,25,36,54]. Surgical intervention is also indicated in the setting of recurrent lymphatic collections or progressive graft dysfunction [54]. Timely diagnosis, surveillance, and management are imperative to avoid graft dysfunction and major complications [54].

## 7. Postoperative Bleeding

Postoperative bleeding requiring surgical intervention, although not high risk in kidney transplant recipients, still warrants careful consideration. Postoperative bleeding most commonly occurs during the early postoperative period, within the first few days after transplantation [13]. It can occur in approximately 4.5% of pediatric kidney transplant recipients, while its incidence in the adult population is 0.2–14% [53]. Surgical site bleeding and hematoma can lead to allograft compression, hemorrhagic shock, and even graft loss [53]. Hematomas are known to be a common early minor postoperative complication that originates from a small leak at a vascular anastomosis or secondary to bleeding from surrounding tissues [21]. Although typically small and asymptomatic, they can increase infection risk, and if large enough, compression can lead to allograft dysfunction and vascular thrombosis [21].

Postoperative bleeding can originate from vascular anastomoses, vessels at the renal hilum or retroperitoneal tissues that were mobilized and exposed during the transplant operation [53]. Hecham et al. reported that the highest risk for postoperative bleeding is within the first 48 h post-kidney transplantation and that factors such as time on dialysis pre-transplant, donor type (deceased donors and expanded criteria donors), lower recipient BMI, and longer cold ischemia time were associated with a statistically significant greater risk of postoperative bleeding in adult kidney transplant recipients [53]. Furthermore, preoperative chronic anticoagulation or antiplatelet therapy was not found to be significantly associated with a greater risk for postoperative bleeding on univariable analysis. Additionally, surgical site bleeding was associated with both an increased risk of long-term graft loss or death in the same population (HR: 1.62, 95% CI: 1.01–2.60, *p* = 0.04).

Ultrasound is the initial imaging modality that detects bleeding and can help differentiate between acute and chronic hematomas [21]. If the recipient remains hemodynamically stable, clinical progression can be monitored with labs, and follow-up abdominal US and CT imaging can be used to assess progression or resolution of the hematoma. Medical management entails red blood cell transfusion and close surveillance of hemodynamic and renal function parameters [13,53]. If concomitant infection is present, percutaneous drainage is warranted [21]. If the patient becomes hemodynamically unstable, if the hematoma is expanding, or if medical management fails, surgical intervention is advised. Hematoma evacuation, identification and control of bleeding sites, and possible vascular anastomotic revision may be required [13,21,53,55].

## 8. Abdominal Compartment Syndrome and Renal Allograft Compartment Syndrome

Abdominal compartment syndrome (ACS) is defined as an intra-abdominal pressure (IAP) ≥ 20 mm Hg with dysfunction of at least one thoracoabdominal organ. An increase in IAP is associated with increased renal vascular resistance and central venous pressure, increased pulmonary artery pressure and pulmonary artery wedge pressure, decreased cardiac output and lung compliance, impaired gas exchange, acidosis, visceral hypoperfusion, and risk of secondary bacterial translocation [55].

A retrospective study from 2014 documented a 2.87% incidence of ACS in pediatric kidney recipients [55]. All pediatric recipients that had ACS were <15 kg at time of transplantation, and all received an adult kidney graft. The most common signs and symptoms were a firm abdomen, hypotension, reduction in ventilation, decrease in lung compliance, increased airway pressure, impaired gas exchange, and associated Doppler ultrasound changes. One patient in this cohort required abdominal exploration and incisional closure with mesh; however, outcomes and mortality rates were not reported. The authors advocate for continuous measurement of urinary bladder pressure in small pediatric patients (<15 kg) who receive a large graft to monitor for the development of ACS. 

Renal allograft compartment syndrome (RACS) is defined as early allograft dysfunction associated with ischemia, secondary to compression of the allograft in the iliac fossa [55,56]. It has not been broadly studied or reported, and in pediatric kidney recipients, its true incidence is unknown, while its incidence in the adult population is reported to be 2% [57]. An increase in iliac fossa pressure over 15–20 mm Hg results in a substantial decrease in renal plasma flow and glomerular filtration rate, and anuria may ensue when pressure exceeds 20 mm Hg [56]. As a result, RACS leads to poor allograft perfusion and can progress to acute tubular injury, renal vascular thrombosis, and graft failure [45]. When an extraperitoneal approach is taken, small pediatric recipients of an adult-sized renal allograft are vulnerable to RACS due to limited extraperitoneal domain [4,58]. Identification of optimal graft location, surgical approach, and vascular reconstruction during transplantation can help mitigate the risk of this complication [8,58].

Management of RACS requires immediate surgical exploration with rapid fascial decompression, assessment of renal vasculature, and/or intraperitoneal repositioning of the graft [56,57]. The use of mesh to aid with a tension-free fascial closure has been proposed; however, data on its use are scarce [55,56,57,59]. Lastly, consideration of skin only closure or systems that aid with wound closure, such as synthetic mesh or negative wound therapy, may be required during the acute phase after a re-exploration for RACS after kidney transplantation [56,57].

## 9. Renal Allograft Torsion

Renal allograft torsion is a rare complication after transplantation. It is caused by the rotation of the transplanted allograft around its vascular pedicle, which significantly alters vascular supply and eventually leads to infarction, necrosis, and graft loss [60,61]. It most commonly presents as an early postoperative complication, but has also been documented 10 years post-kidney transplantation [60,62]. Few cases of renal allograft torsion have been reported in both adult and pediatric populations; however, all cases in children have led to graft nephrectomy, unlike what has been reported in adults [60]. In adults, mechanisms explaining renal allograft torsion include allograft vein, artery, and ureter length, increased space and hypermobility of an intraperitoneally placed graft, abdominal wall laxity, and decreased or minimal adhesion formation associated with immunosuppressant use [60]. More specifically, mTOR inhibitors, like sirolimus, inhibit fibrosis and wound healing and can lead to an adhesion-free environment facilitating the free rotation of the allograft [63]. Non-specific signs and symptoms, such as decreased renal function, abdominal pain, oliguria, and vomiting, can ensue, mimicking other complications, such as acute tubular necrosis, acute rejection, ureteral obstruction, and renal vein thrombosis [61,63].

**Figure 7:**
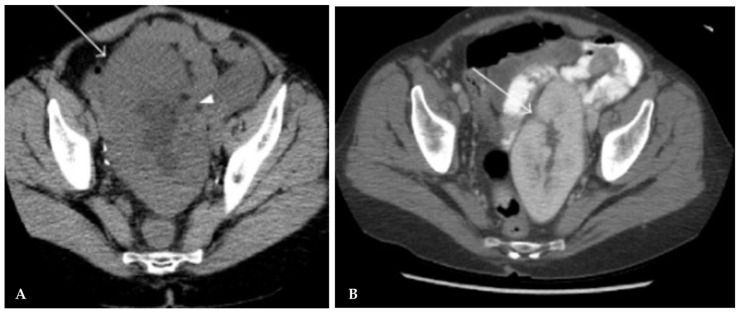
Non-contrast (**A**) and contrast enhanced (**B**) CT scan of the abdomen and pelvis. (**A**) Enlarged left lower quadrant renal allograft (arrow) with hilum directed laterally (arrowhead). (**B**) Previous imaging with an anteromedially directed hilum (arrow). Image reused from “Renal Allograft Torsion: US and CT Imaging Findings of a Rare Posttransplant Complication”, by Dewan S et al., published by Case Reports in Radiology, accessed on 18 December 2025, licensed under CC BY 4.0 (http://creativecommons.org/licenses/by/4.0/) [64]. Minor modifications were made to the title and descriptive captions. The background and size of the figure were also adjusted from the original source.

The diagnosis of renal allograft torsion requires a high level of suspicion. However, it is not uncommon to make the definitive diagnosis intraoperatively [61]. Notably, timely diagnosis can be challenging due to the lack of specific ultrasound findings during an incomplete torsion, or during the early stages of a complete torsion [61]. Color Doppler ultrasound can identify concerning, but not specific, features related to arterial inflow and resistive indices. However, reversal of diastolic flow and severe reduction or complete absence of intrarenal blood flow may only appear with severe and irreversible damage [61]. CT with or without contrast can help identify a change of axis (Figure 7), in comparison to previous imaging, yet CT is not always telling if there are no longitudinal discrepancies [61]. If suspected, emergent re-exploration is indicated to assess the graft and the vascular pedicle [61]. Detorsion and confirmation of correct anatomic positioning of the graft, ureter, and pedicle is necessary to assess graft viability and ultimately decide whether graft nephrectomy is required [62]. Some authors favor the use of nephropexy to avoid repeat torsion of the kidney [63]; however, its role in preventing future torsion is unclear [60,63] [63]. A literature review by Lucewicz et al. identified 16 cases of intraperitoneal renal allograft torsion in adults, of which 13 were in the setting of simultaneous kidney-pancreas transplantation [63]. Of these 16 cases, 44% were salvaged, 38% had immediate nephrectomy, and 29% were acutely salvaged, but subsequently required nephrectomy. Although a rare postoperative complication, high clinical suspicion and immediate reoperation are critical to avoid graft nephrectomy [61,63].

## 10. Discussion

Despite the relatively low incidence of graft loss and post-kidney transplant complications, well-designed prospective studies are needed to optimize surgical practice and prophylactic techniques. As a result of scarce evidence-based standardized surgical practices for children undergoing kidney transplantation, surgical technique, postoperative monitoring and even prophylactic measures, vary greatly amongst institutions. Currently, pediatric specific recommendations are absent from the 2024 European Association of Urology Guidelines on Renal Transplantation [65]. The Kidney Disease: Improving Global Outcomes (KDIGO) 2020 guideline has a brief section covering pediatric issues, in which only presurgical neurocognitive and academic assessments prior to transplantation in children are addressed [66]. However, a non-graded recommendation, non-specific to children, for presurgical cross-sectional imaging of the upper and/or lower extremities to confirm vascular patency in patients with extensive surgical history or previous hemodialysis is issued, as vascular access is affected and may alter the surgical planning [66]. The guidelines advocate for a urologist with experience in transplantation to help navigate care in patients with increased risk, such as those with significant urologic structural abnormalities or dysfunctional voiding. The 2024 European Association of Urology Guidelines on Renal Transplantation acknowledges the absence of thromboprophylaxis guidelines during the perioperative period and advocates for the use of anticoagulation on a case-by-case basis [65]. A specific section on technical recommendations, although not tailored to the pediatric population, is included. Recommendations include ligation of peri-iliac vessel lymphatics to decrease the risk of postoperative lymphocele, and the use of cooling of the kidney surface during implantation. Additionally, the importance of renal artery and vein length is highlighted to avoid kinking. Finally, an extravesical ureteroneocystostomy anastomosis with ureteral stent is the elected technique to minimize urinary tract complications in recipients with normal urologic anatomy.

Despite general guidance on some aspects of surgical technique, there is a notable absence of comprehensive and inclusive surgical standards addressing kidney transplantation in children. Specific techniques related to ureteral implantation and the use of stents remain to be standardized. As transplantation in children presents unique challenges, focused efforts are needed to define optimal perioperative strategies and to standardize surgical care. Furthermore, establishing best practices for postoperative surveillance, thromboprophylaxis, and imaging protocols are crucial to facilitating early detection and improving allograft salvage rates and long-term viability. With the advent of surgical innovation, data on endoscopic and minimally invasive modalities in children are also needed.

While this narrative review provides an overview of the existing literature on the management of surgical complications following kidney transplantation in children, it is important to acknowledge its limitations. A narrative review is inherently subject to selection bias, as the inclusion of articles is not pre-defined by a systemic search strategy or a pre-determined protocol. Moreover, as this is a descriptive study, the lack of quantitative analysis limits its interpretation and the generalizability of our findings. Finally, due to the paucity of evidence specific to children, data is almost exclusively from observational studies, as prospective studies are lacking in the majority of these complications. This poses notable constraints on the strength and generalizability of our findings.

Future multicentric studies evaluating long-term outcomes on specific surgical techniques, postoperative surveillance, and treatment modalities will be pivotal to optimize clinical outcomes in this population. Finally, interdisciplinary collaborations have the potential to capture a greater population and therefore advance the development of surgical standards of care.

## 11. Conclusions and Future Perspectives

Although pediatric kidney transplant experience has increased and outcomes are comparable to adult kidney transplantation, surgical complications in children still pose a significant threat to graft viability and lead to poorer outcomes. Pediatric kidney transplant recipients pose specific challenges related to their underlying disease and anatomic size discrepancies that ultimately require meticulous surgical planning and careful intraoperative considerations. Surgical technique and intraoperative efforts are the ultimate prevention and can be definitive in decreasing the incidence of complications, morbidity, and mortality. Furthermore, clinical acumen and low index of suspicion for complications and early intervention can drastically change the clinical course and mitigate the need for graft nephrectomy. Standardized protocols aimed at implementing intraoperative and early postoperative clinical and imaging tools to efficiently diagnose the most common and threatening complications can mitigate the risk of irreversible allograft damage and allograft loss. Multicenter prospective pediatric studies are needed to further elucidate the risks and outcomes of surgical complications in children and to optimize care algorithms focused on decreasing graft loss and increasing long-term graft function in pediatric kidney transplant recipients.

## Data Availability

This review article does not have any primary data to make available.

## Abbreviations

The following abbreviations are used in this manuscript:

ACS	Abdominal compartment syndrome
ASA	Acetylsalicylic acid
BK	Polyomavirus BK
BMI	Body mass index
CMV	Cytomegalovirus
CT	Computed tomography
CVP	Central venous pressure
ESRD	End stage renal disease
IAP	Intra-abdominal pressure
MR	Magnetic Resonance
OPTN	Organ Procurement and Transplantation Network
RACS	Renal allograft compartment syndrome
SSI	Surgical site infection
UTI	Urinary tract infection
VT	Vascular thrombosis
VUR	Vesicoureteral reflux
